# Bis{*N*-[2-hy­droxy-1,1-bis­(hy­droxy­methyl)eth­yl]glycinato-κ^3^ *O*,*N*,*O*′}iron(II)

**DOI:** 10.1107/S160053681401397X

**Published:** 2014-06-21

**Authors:** Ning Jiang, Juan-Juan Hou

**Affiliations:** a School of Chemistry and Material Science, Shanxi Normal University, Linfen 041004, People’s Republic of China

**Keywords:** crystal structure

## Abstract

In the title compound, [Fe(C_6_H_12_NO_5_)_2_], the Fe^II^ ion lies on an inversion center and is coordinated by two N atoms and four O atoms from two tridentate *N*-[2-hy­droxy-1,1-bis­(hy­droxy­methyl)eth­yl]glycine ligands, forming a slightly distorted octa­hedral coordination environment. In the crystal, O—H⋯O, O—H⋯N and weak C—H⋯O hydrogen bonds link mol­ecules, forming a three-dimensional network.

## Related literature   

For background to the applications of tripodal alcohols as single-mol­ecule magnets, see: Pilawa *et al.* (1998 ▶); Brechin (2005 ▶); Murugesu *et al.* (2005 ▶). 
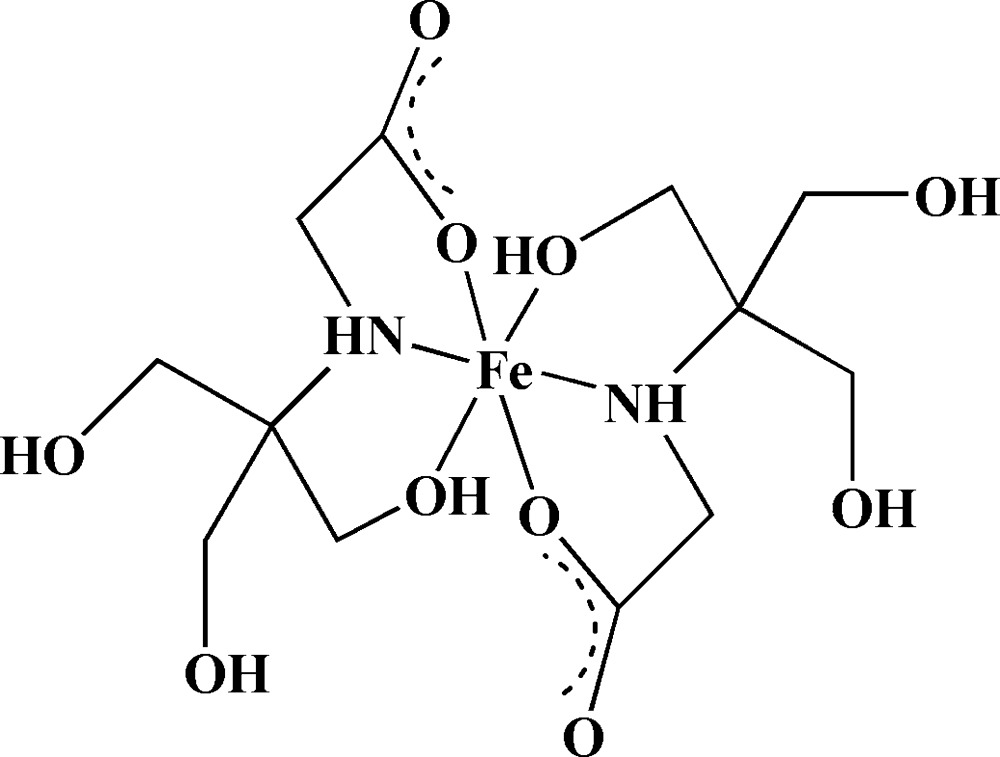


## Experimental   

### 

#### Crystal data  

[Fe(C_6_H_12_NO_5_)_2_]
*M* *_r_* = 412.18
Monoclinic, 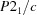

*a* = 8.8198 (7) Å
*b* = 9.0245 (7) Å
*c* = 12.3533 (7) Å
β = 127.224 (4)°
*V* = 782.94 (10) Å^3^
*Z* = 2
Mo *K*α radiation
μ = 1.02 mm^−1^
*T* = 298 K
0.19 × 0.16 × 0.08 mm

#### Data collection  

Bruker SMART CCD diffractometer
Absorption correction: multi-scan (*SADABS*; Bruker, 2007 ▶) *T*_min_ = 0.829, *T*_max_ = 0.923
4000 measured reflections
1708 independent reflections
1230 reflections with *I* > 2σ(*I*)
*R*_int_ = 0.056

#### Refinement  

*R*[*F* ^2^ > 2σ(*F* ^2^)] = 0.046
*wR*(*F* ^2^) = 0.099
*S* = 1.04
1708 reflections
131 parameters
4 restraints
H atoms treated by a mixture of independent and constrained refinement
Δρ_max_ = 0.46 e Å^−3^
Δρ_min_ = −0.39 e Å^−3^

### 

Data collection: *SMART* (Bruker, 2007 ▶); cell refinement: *SAINT-Plus* (Bruker, 2007 ▶); data reduction: *SAINT-Plus*; program(s) used to solve structure: *SHELXS97* (Sheldrick, 2008 ▶); program(s) used to refine structure: *SHELXL97* (Sheldrick, 2008 ▶); molecular graphics: *DIAMOND* (Brandenburg & Putz, 2006 ▶); software used to prepare material for publication: *SHELXTL* (Sheldrick, 2008 ▶).

## Supplementary Material

Crystal structure: contains datablock(s) I, New_Global_Publ_Block. DOI: 10.1107/S160053681401397X/lh5715sup1.cif

Structure factors: contains datablock(s) I. DOI: 10.1107/S160053681401397X/lh5715Isup2.hkl

Supporting information file. DOI: 10.1107/S160053681401397X/lh5715Isup3.cdx

Additional supporting information:  crystallographic information; 3D view; checkCIF report

## Figures and Tables

**Table 1:** Hydrogen-bond geometry (Å, °)

*D*—H⋯*A*	*D*—H	H⋯*A*	*D*⋯*A*	*D*—H⋯*A*
N1—H1*A*⋯O2^i^	0.87 (2)	2.10 (2)	2.952 (4)	166 (3)
O3—H3*A*⋯O4^ii^	0.86 (2)	1.72 (2)	2.562 (4)	167 (5)
O1—H1*B*⋯O5^iii^	0.85 (2)	1.96 (2)	2.804 (4)	174 (6)
O1—H1*B*⋯O4^iii^	0.85 (2)	2.59 (5)	3.172 (4)	127 (4)
O2—H2*C*⋯O1^ii^	0.85 (2)	1.93 (2)	2.779 (4)	170 (5)
C5—H5*B*⋯O5^iv^	0.97	2.56	3.452 (4)	153
C2—H2*A*⋯O1	0.97	2.56	3.184 (4)	122

Symmetry codes: (i) 
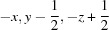
; (ii) 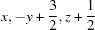; (iii) 
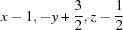
; (iv) 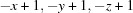.

**Fig. 1.**
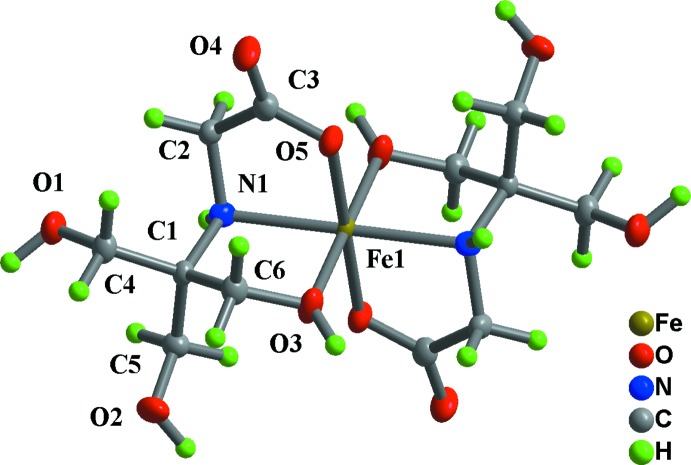
The molecular structure of the title compound showing 50% displacement ellipsoids. Unlabeled atoms are related by the symmetry operator (-x+1, -y+1, -z+1).

## supplementary crystallographic information

### S1. Comment

Tripodal alcohols have been used as poly-dentate ligands in combination with paramagnetic 3d transition metal ions leading to the formation of high nuclear clusters since the discovery of the phenomenon of single-molecule magnetism (Brechin, 2005; Murugesu *et al.*, 2005; Pilawa *et al.*, 1998). During our synthesis to form a poly-nuclear cluster using the *N*-[tris(hydroxymethyl)ethyl]glycine ligand the title compound was fortuitously obtained.

In the title molecule the Fe^II^ ion is located on an inversion center (Fig. 1). The Fe^II^ ion is in a slightly distorted octahedral coordination environment formed by two N atoms and four O atoms from two *N*-[tris(hydroxymethyl)ethyl]glycine ligands. In the crystal, classical O—H···O, O—H···N and weak C—H···O hydrogen bonds (Table 1) connect the molecules into a three-dimensional superamolecular architecture.

### S2. Experimental

The title compound was synthesized hydrothermally under autogenous pressure. A mixture of FeSO_4_ (0.028 g, 0.1 mmol), *N*-[tris(hydroxymethyl)ethyl]glycine (0.056 g, 0.3 mmol), methanol (3 ml), N,*N*'-dimethyl formamide (1 ml) and H_2_O (2 ml), was stirred for 30 min and then sealed in a 15 ml Teflon-lined stainless container and heated to 358K for 60 h. After cooling to room temperature and subjected to filltration, colorless plates were recovered.

### S3. Refinement

Hydrogen atoms bonded to N and O atoms were located in a difference map and refined with distance restraints of O—H = 0.84 (2) and N—H = 0.87 (2) Å. Other H atoms were positioned geometrically and refined using a riding model with C—H = 0.95–0.99 Å.

### Crystal data

**Table:**

[Fe(C_6_H_12_NO_5_)_2_]	*F*(000) = 432
*M**_r_* = 412.18	*D*_x_ = 1.748 Mg m^−^^3^
Monoclinic, *P*2_1_/*c*	Mo *K*α radiation, λ = 0.71073 Å
Hall symbol: -P 2ybc	Cell parameters from 621 reflections
*a* = 8.8198 (7) Å	θ = 2.9–21.9°
*b* = 9.0245 (7) Å	µ = 1.02 mm^−^^1^
*c* = 12.3533 (7) Å	*T* = 298 K
β = 127.224 (4)°	Sheet, colorless
*V* = 782.94 (10) Å^3^	0.19 × 0.16 × 0.08 mm
*Z* = 2	

### Data collection

**Table:**

Bruker SMART CCD diffractometer	1708 independent reflections
Radiation source: fine-focus sealed tube	1230 reflections with *I* > 2σ(*I*)
Graphite monochromator	*R*_int_ = 0.056
multi–scan	θ_max_ = 27.0°, θ_min_ = 2.9°
Absorption correction: multi-scan (*SADABS*; Bruker, 2007)	*h* = −11→11
*T*_min_ = 0.829, *T*_max_ = 0.923	*k* = −11→11
4000 measured reflections	*l* = −15→15

### Refinement

**Table:**

Refinement on *F*^2^	Primary atom site location: structure-invariant direct methods
Least-squares matrix: full	Secondary atom site location: difference Fourier map
*R*[*F*^2^ > 2σ(*F*^2^)] = 0.046	Hydrogen site location: inferred from neighbouring sites
*wR*(*F*^2^) = 0.099	H atoms treated by a mixture of independent and constrained refinement
*S* = 1.04	*w* = 1/[σ^2^(*F*_o_^2^) + (0.034*P*)^2^] where *P* = (*F*_o_^2^ + 2*F*_c_^2^)/3
1708 reflections	(Δ/σ)_max_ < 0.001
131 parameters	Δρ_max_ = 0.46 e Å^−^^3^
4 restraints	Δρ_min_ = −0.39 e Å^−^^3^

### Special details

**Table:**

Geometry. All e.s.d.'s (except the e.s.d. in the dihedral angle between two l.s. planes) are estimated using the full covariance matrix. The cell e.s.d.'s are taken into account individually in the estimation of e.s.d.'s in distances, angles and torsion angles; correlations between e.s.d.'s in cell parameters are only used when they are defined by crystal symmetry. An approximate (isotropic) treatment of cell e.s.d.'s is used for estimating e.s.d.'s involving l.s. planes.
Refinement. Refinement of *F*^2^ against ALL reflections. The weighted *R*-factor *wR* and goodness of fit *S* are based on *F*^2^, conventional *R*-factors *R* are based on *F*, with *F* set to zero for negative *F*^2^. The threshold expression of *F*^2^ > σ(*F*^2^) is used only for calculating *R*-factors(gt) *etc*. and is not relevant to the choice of reflections for refinement. *R*-factors based on *F*^2^ are statistically about twice as large as those based on *F*, and *R*- factors based on ALL data will be even larger.

### Fractional atomic coordinates and isotropic or equivalent isotropic displacement parameters (Å^2^)

**Table:**

	*x*	*y*	*z*	*U*_iso_*/*U*_eq_	
Fe1	0.5000	0.5000	0.5000	0.01527 (19)	
O1	−0.0265 (4)	0.7341 (3)	0.0516 (3)	0.0347 (7)	
O2	0.0173 (4)	0.8369 (3)	0.3530 (3)	0.0355 (7)	
O3	0.4719 (4)	0.7180 (3)	0.5354 (3)	0.0323 (7)	
O4	0.6437 (4)	0.6393 (3)	0.2600 (3)	0.0356 (7)	
O5	0.6619 (3)	0.5743 (3)	0.4408 (2)	0.0283 (6)	
N1	0.2766 (4)	0.5686 (3)	0.2962 (3)	0.0209 (6)	
C1	0.2121 (5)	0.7215 (4)	0.2983 (3)	0.0190 (8)	
C2	0.3608 (5)	0.5508 (4)	0.2233 (3)	0.0237 (8)	
H2A	0.2927	0.6132	0.1430	0.028*	
H2B	0.3466	0.4488	0.1940	0.028*	
C3	0.5700 (5)	0.5920 (4)	0.3128 (4)	0.0243 (8)	
C4	0.1289 (5)	0.8101 (4)	0.1682 (4)	0.0276 (9)	
H4A	0.0855	0.9056	0.1758	0.033*	
H4B	0.2274	0.8276	0.1572	0.033*	
C5	0.0626 (5)	0.6982 (4)	0.3222 (4)	0.0269 (8)	
H5A	−0.0516	0.6556	0.2416	0.032*	
H5B	0.1112	0.6298	0.3971	0.032*	
C6	0.3822 (5)	0.8077 (4)	0.4165 (4)	0.0269 (8)	
H6A	0.4712	0.8302	0.3973	0.032*	
H6B	0.3396	0.9003	0.4299	0.032*	
H1A	0.182 (4)	0.506 (3)	0.259 (3)	0.027 (10)*	
H3A	0.514 (6)	0.773 (5)	0.605 (3)	0.078 (18)*	
H1B	−0.126 (5)	0.787 (5)	0.014 (5)	0.10 (2)*	
H2C	0.019 (7)	0.815 (5)	0.421 (4)	0.080 (19)*	

### Atomic displacement parameters (Å^2^)

**Table:**

	*U*^11^	*U*^22^	*U*^33^	*U*^12^	*U*^13^	*U*^23^
Fe1	0.0159 (4)	0.0181 (3)	0.0101 (4)	0.0003 (3)	0.0069 (3)	0.0009 (3)
O1	0.0265 (16)	0.0462 (18)	0.0188 (15)	0.0048 (14)	0.0070 (14)	0.0003 (13)
O2	0.0443 (18)	0.0349 (16)	0.0402 (19)	0.0148 (13)	0.0323 (16)	0.0086 (14)
O3	0.0379 (17)	0.0292 (15)	0.0160 (15)	0.0004 (13)	0.0091 (14)	−0.0017 (12)
O4	0.0293 (15)	0.0513 (18)	0.0283 (16)	0.0014 (13)	0.0185 (14)	0.0135 (13)
O5	0.0234 (14)	0.0396 (15)	0.0174 (14)	0.0003 (12)	0.0100 (12)	0.0048 (12)
N1	0.0203 (16)	0.0195 (16)	0.0220 (17)	−0.0026 (13)	0.0123 (15)	−0.0040 (13)
C1	0.0192 (18)	0.0201 (18)	0.0148 (19)	0.0020 (14)	0.0087 (16)	0.0019 (14)
C2	0.0213 (19)	0.0286 (19)	0.018 (2)	0.0028 (15)	0.0103 (17)	−0.0004 (15)
C3	0.026 (2)	0.0218 (19)	0.024 (2)	0.0015 (16)	0.0141 (18)	0.0035 (16)
C4	0.027 (2)	0.029 (2)	0.022 (2)	0.0025 (17)	0.0123 (19)	0.0029 (17)
C5	0.027 (2)	0.031 (2)	0.025 (2)	0.0050 (17)	0.0166 (19)	0.0038 (17)
C6	0.025 (2)	0.027 (2)	0.023 (2)	−0.0003 (16)	0.0121 (18)	−0.0005 (17)

### Geometric parameters (Å, º)

**Table:**

Fe1—O3^i^	2.062 (3)	N1—C1	1.498 (4)
Fe1—O3	2.062 (3)	N1—H1A	0.871 (18)
Fe1—O5^i^	2.071 (2)	C1—C4	1.527 (4)
Fe1—O5	2.071 (2)	C1—C6	1.529 (5)
Fe1—N1	2.145 (3)	C1—C5	1.531 (4)
Fe1—N1^i^	2.145 (3)	C2—C3	1.515 (5)
O1—C4	1.427 (4)	C2—H2A	0.9700
O1—H1B	0.850 (19)	C2—H2B	0.9700
O2—C5	1.433 (4)	C4—H4A	0.9700
O2—H2C	0.854 (19)	C4—H4B	0.9700
O3—C6	1.426 (4)	C5—H5A	0.9700
O3—H3A	0.855 (19)	C5—H5B	0.9700
O4—C3	1.242 (4)	C6—H6A	0.9700
O5—C3	1.277 (4)	C6—H6B	0.9700
N1—C2	1.482 (4)		
			
O3^i^—Fe1—O3	180.000 (1)	N1—C1—C5	104.9 (3)
O3^i^—Fe1—O5^i^	87.88 (10)	C4—C1—C5	110.7 (3)
O3—Fe1—O5^i^	92.12 (10)	C6—C1—C5	110.3 (3)
O3^i^—Fe1—O5	92.12 (10)	N1—C2—C3	111.5 (3)
O3—Fe1—O5	87.88 (10)	N1—C2—H2A	109.3
O5^i^—Fe1—O5	180.0	C3—C2—H2A	109.3
O3^i^—Fe1—N1	99.75 (10)	N1—C2—H2B	109.3
O3—Fe1—N1	80.25 (10)	C3—C2—H2B	109.3
O5^i^—Fe1—N1	99.68 (10)	H2A—C2—H2B	108.0
O5—Fe1—N1	80.32 (10)	O4—C3—O5	123.4 (3)
O3^i^—Fe1—N1^i^	80.25 (10)	O4—C3—C2	119.6 (3)
O3—Fe1—N1^i^	99.75 (10)	O5—C3—C2	117.0 (3)
O5^i^—Fe1—N1^i^	80.32 (10)	O1—C4—C1	111.5 (3)
O5—Fe1—N1^i^	99.68 (10)	O1—C4—H4A	109.3
N1—Fe1—N1^i^	180.000 (1)	C1—C4—H4A	109.3
C4—O1—H1B	109 (4)	O1—C4—H4B	109.3
C5—O2—H2C	102 (3)	C1—C4—H4B	109.3
C6—O3—Fe1	112.7 (2)	H4A—C4—H4B	108.0
C6—O3—H3A	109 (3)	O2—C5—C1	110.0 (3)
Fe1—O3—H3A	137 (3)	O2—C5—H5A	109.7
C3—O5—Fe1	114.9 (2)	C1—C5—H5A	109.7
C2—N1—C1	116.4 (3)	O2—C5—H5B	109.7
C2—N1—Fe1	103.9 (2)	C1—C5—H5B	109.7
C1—N1—Fe1	109.5 (2)	H5A—C5—H5B	108.2
C2—N1—H1A	106 (2)	O3—C6—C1	107.9 (3)
C1—N1—H1A	111 (2)	O3—C6—H6A	110.1
Fe1—N1—H1A	110 (2)	C1—C6—H6A	110.1
N1—C1—C4	114.2 (3)	O3—C6—H6B	110.1
N1—C1—C6	108.8 (3)	C1—C6—H6B	110.1
C4—C1—C6	107.9 (3)	H6A—C6—H6B	108.4
			
O5^i^—Fe1—O3—C6	−120.1 (2)	Fe1—N1—C1—C6	32.2 (3)
O5—Fe1—O3—C6	59.9 (2)	C2—N1—C1—C5	156.7 (3)
N1—Fe1—O3—C6	−20.6 (2)	Fe1—N1—C1—C5	−85.9 (3)
N1^i^—Fe1—O3—C6	159.4 (2)	C1—N1—C2—C3	83.5 (4)
O3^i^—Fe1—O5—C3	84.2 (2)	Fe1—N1—C2—C3	−37.0 (3)
O3—Fe1—O5—C3	−95.8 (2)	Fe1—O5—C3—O4	178.2 (3)
N1—Fe1—O5—C3	−15.3 (2)	Fe1—O5—C3—C2	−2.4 (4)
N1^i^—Fe1—O5—C3	164.7 (2)	N1—C2—C3—O4	−152.0 (3)
O3^i^—Fe1—N1—C2	−62.5 (2)	N1—C2—C3—O5	28.6 (4)
O3—Fe1—N1—C2	117.5 (2)	N1—C1—C4—O1	56.5 (4)
O5^i^—Fe1—N1—C2	−152.0 (2)	C6—C1—C4—O1	177.6 (3)
O5—Fe1—N1—C2	28.0 (2)	C5—C1—C4—O1	−61.6 (4)
O3^i^—Fe1—N1—C1	172.4 (2)	N1—C1—C5—O2	168.6 (3)
O3—Fe1—N1—C1	−7.6 (2)	C4—C1—C5—O2	−67.7 (4)
O5^i^—Fe1—N1—C1	82.9 (2)	C6—C1—C5—O2	51.6 (4)
O5—Fe1—N1—C1	−97.1 (2)	Fe1—O3—C6—C1	43.9 (3)
C2—N1—C1—C4	35.4 (4)	N1—C1—C6—O3	−49.6 (3)
Fe1—N1—C1—C4	152.8 (2)	C4—C1—C6—O3	−174.0 (3)
C2—N1—C1—C6	−85.3 (3)	C5—C1—C6—O3	65.0 (3)

Symmetry code: (i) −*x*+1, −*y*+1, −*z*+1.

### Hydrogen-bond geometry (Å, º)

**Table:**

*D*—H···*A*	*D*—H	H···*A*	*D*···*A*	*D*—H···*A*
N1—H1*A*···O2^ii^	0.87 (2)	2.10 (2)	2.952 (4)	166 (3)
O3—H3*A*···O4^iii^	0.86 (2)	1.72 (2)	2.562 (4)	167 (5)
O1—H1*B*···O5^iv^	0.85 (2)	1.96 (2)	2.804 (4)	174 (6)
O1—H1*B*···O4^iv^	0.85 (2)	2.59 (5)	3.172 (4)	127 (4)
O2—H2*C*···O1^iii^	0.85 (2)	1.93 (2)	2.779 (4)	170 (5)
C5—H5*B*···O5^i^	0.97	2.56	3.452 (4)	153
C2—H2*A*···O1	0.97	2.56	3.184 (4)	122

Symmetry codes: (i) −*x*+1, −*y*+1, −*z*+1; (ii) −*x*, *y*−1/2, −*z*+1/2; (iii) *x*, −*y*+3/2, *z*+1/2; (iv) *x*−1, −*y*+3/2, *z*−1/2.
